# Comparison of the value of PCNA and Ki-67 as markers of cell proliferation in ameloblastic tumor

**DOI:** 10.4317/medoral.18573

**Published:** 2012-12-10

**Authors:** Ronell Bologna-Molina, Adalberto Mosqueda-Taylor, Nelly Molina-Frechero, Ana D. Mori-Estevez, Guillermo Sánchez-Acuña

**Affiliations:** 1 Research Department, School of Dentistry, Universidad Juárez del Estado de Durango (UJED), Durango, México; 2 School of Dentistry, Universidad de la República (UDELAR). Montevideo, Uruguay; 3 Health Care Department, Universidad Autónoma Metropolitana, Xochimilco. Mexico City, Mexico; 4 Pathology Department , Hospital Universitario “Gral. Calixto García” La Havana, Cuba; 5 Maxillofacial Department, Hospital Universitario “Gral. Calixto García” La Havana, Cuba

## Abstract

Objectives: The aim of this study was to compare among PCNAand Ki-67 as the most reliable immunohistochemical marker for evaluating cell proliferation in ameloblastic tumors. Study Design: Observational, retrospective, and descriptive study of a large series of ameloblastic tumors, composed of 161 ameloblastomas and four ameloblastic carcinomas, to determine and compare PCNA and Ki-67 expression using immunohistochemistry techniques. Results: When analyzing Ki-67 positivity, the desmoplastic ameloblastoma demonstrated a significantly lower proliferation rate (1.9%) compared with the solid/multicystic and unicystic ameloblastomas and ameloblastic carcinomas (p<0.05), whereas the ameloblastic carcinomas displayed a significantly higher rate compared with all of the other ameloblastomas (48.7%) (p<0.05). When analyzing cell proliferation with PCNA, we found significant differences only between the ameloblastic carcinomas (93.3%) and the desmoplastic ameloblastomas (p<0.05). When differences between the immunopositivity for PCNA and Ki-67 were compared, the percentages were higher for PCNA in all types of ameloblastomas and ameloblastic carcinomas. In all cases, the percentages were greater than 80%, whereas the immunopositivity for Ki-67 was significantly lower; for example, the ameloblastic carcinoma expressed the highest positivity and only reached 48.7%, compared to 93.3% when we used PCNA. Conclusions: In the present study, when we used the proliferation cell marker Ki-67, the percentages of positivity were more specific and varied among the different types of ameloblastomas, suggesting that Ki-67 is a more specific marker for the proliferation of ameloblastic tumor cells.

** Key words:**Ameloblastomas, ameloblastic carcinoma, PCNA, Ki-67, cell proliferation markers.

## Introduction

Cell proliferation is a biological process that is essential to all living organisms due to its role in the growth and maintenance of tissue homeostasis (1). The control of this important process is completely dysregulated in some types of neoplasias (2,3), and the assessment of cell proliferation activity in tumors has become a common tool used by histopathologists to provide useful information for assessing diagnosis, clinical behavior, and therapy.

The cell cycle consists of a series of phases, during which there are changes that lead to cell division. Cell regulatory genes modulate the cell cycle in a highly sophisticated fashion via a number of proteins (4).

Proliferation markers refers to specific proteins or other factors whose presence in actively growing and dividing cells serves as an indicator for such cells.

Today, the most common method for determining proliferative activity is the use of immunohistochemical techniques, which are increasingly being applied in routine pathology.

The Ki-67 antigen (Ki-67) is a classic marker of cellular proliferation that has been widely applied in the diagnostic, research and drug-discovery fields. The Ki-67 antigen was originally defined by the monoclonal antibody Ki-67, with the name derived from the city of origin (Kiel) and the number of the original clone in the 96-well plate (5). The monoclonal antibody Ki67 was first described in 1983 by Johannes Gerdes and colleagues, who suggested that it might be used as a marker for proliferating cells (5).

The Ki-67 antigen is preferentially expressed during the late G1, S, G2 and M phase of the cell cycle, whereas resting, non-cycling cells (G0 phase) lack Ki-67 expression. Because of its absence in quiescent cells (G0 phase), this protein developed into a widely used tumor marker in the fields of research and pathology. The standard antibody for the detection of Ki-67 is MIB-1. The fraction of MIB-1-positive tumor cells (the MIB-1/Ki-67 labeling index) is often correlated with the clinical course of cancer; Ki-67is of prognostic value for many types of malignant tumors (6).

Proliferating Cell Nuclear Antigen (PCNA) is a nuclear nonhistone protein that is necessary for DNA synthesis and is an accessory protein for DNA polymerase alpha, which is elevated during the G1/S phase of the cell cycle. Quiescent and senescent cells have very low levels of PCNA mRNA (7). PCNA expression may be used as a marker of cell proliferation because cells remain a longer time in the G1/S phase when proliferating. Furthermore, this protein has an essential role in nucleic acid metabolism as a component of the DNA replication and repair mechanism (8).

An increase in PCNA levels may be induced by growth factors or as a result of DNA damage in the absence of cell cycling (8,9).

PCNA is an essential factor for DNA replication and repair. PCNA forms a toroidal, ring-shaped structure of 90 kDa by the symmetric association of three identical monomers. The ring encircles the DNA, acts as a platform upon which polymerases and other proteins dock to perform various DNA metabolic processes, and functions as a DNA polymerase-delta co-factor (10).

Various cell proliferation markers have been used in several studies as diagnostic and prognostic tools as well as aids in understanding the biological behavior in many stages of disease. Currently, new markers are being added to evaluate cell proliferation. However, PCNA is still used as a marker of cell proliferation, and Ki-67 is considered the classic marker of cell proliferation and is in routine use by pathologists. Furthermore, several studies have been performed to evaluate cell proliferation using PCNA and Ki-67 in different tumors of various origins; compared with PCNA, Ki-67 has been shown to be more sensitive and specific in the various tumors analyzed (11-13).

Despite the existence of these data, there are still numerous studies using PCNA as the first-choice marker of cell proliferation (13-15). Many investigations of tumor-cell proliferative activity have used PCNA and Ki-67 to evaluate cell proliferation in oral tumors (16-18). The aim of this study was to determine which of the two markers is more useful to evaluate cell proliferation in the study of ameloblastic tumors.

## Material and Methods

Four ameloblastic carcinomas and 161 ameloblastomas from the files of the Laboratory of Oral Pathology of the Universidad Autónoma Metropolitana Xochimilco (Mexico City, Mexico), a private oral pathology service in Mexico City and the Pathology Department of the Hospital “Calixto Garcia” (La Havana, Cuba) were included. In all cases diagnosis was established according the criteria of the current W.H.O. Histological Classification of Tumours (19).

The paraffin blocks were sliced into 2 µm thick sections, and the tissue sections were mounted on poly lysine-coated glass slides and air-dried overnight at room temperature. After deparaffinization and rehydration, the tissue sections were treated with 0.1 M sodium citrate (pH 6.2) and Tween 20 to unravel the epitopes. Endogenous peroxidases were blocked with 0.9% hydrogen peroxide, followed by incubation with 1% bovine serum albumin in PBS for 5 min to eliminate nonspecific binding. Monoclonal antibodies were used against Ki-67 (clone MIB-1; 1:100 dilution, Dako, Carpinteria, CA, USA) and PCNA (Clone PC10; dilution 1:100, Dako, Carpinteria, CA, USA). The sections were incubated with primary antibodies for 45 min; subsequently, the sections were incubated with a biotinylated anti-mouse/anti-rabbit antibody and with the streptavidin/peroxidase complex for 30 min each (LSAB þ-labeled streptavidin-biotin, Dako). A 3,30-diaminobenzidine-H2O (Dako) substrate was used to visualize the reaction. Subsequently, the sections were counterstained with Mayer’s hematoxylin solution. For the negative controls, the primary antibody was replaced with PBS. The evaluation of Ki-67 and PCNA staining was performed using selected areas that were rich in epithelial cells from each case of solid ameloblastomas and along the cyst lining and mural follicles of the unicystic variant. All of the cell counts were performed as previously described (20). The labeling index (number of positive tumor cells/total number of tumor cells expressed as a percentage) was calculated in every case.

Statistical analysis was performed using the chi square, Pearson’s or Fisher’s Exact tests, considering the expected values. The Mann–Whitney test (U-test) for differences between the two groups or the Kruskal–Wallis test for differences among three or more groups was used to detect differences between the various types of lesions and the PCNA and Ki-67 indicators and Pearson correlation factor for analysis the correlation between the PCNA and Ki-67. The results were considered significant for p≤0.05. The results were analyzed using the SPSS 16.0 statistical software (SPSS Professional Statistics, SPSS Inc., Chicago, IL).

## Results

In the present study, 87 out of 161 cases of ameloblastomaswere classified as unicystic (UA), including 46 cases of the intralu-minal variant (IUA), 26 cases of the mural variant (MUA) and 15 cases of the simple/luminal variant (LUA). There were 66 cases of solid/multicysticameloblastoma (SA), of which there were 35 plexiform (PSA), 14 follicular (FSA), 15 acanthomatous (ASA) and two basal cell types (BSA). There were also five desmoplastic (DA) and three peripheral ameloblastomas (PA). In addition, there were four ameloblastic carcinomas (AC).

When we analyzed the positivity for Ki-67 (using the labeling index), there were significant differences between the various types of ameloblastomas. The DA displayed a significantly lower proliferation rate (1.9%) compared with the rest of the central ameloblastomas and the ACs (p<0.05), whereas the AC demonstrated a significantly higher rate compared with the other benign ameloblastomas (48.7%) (p<0.05) ( Table 1).

**Table 1:**
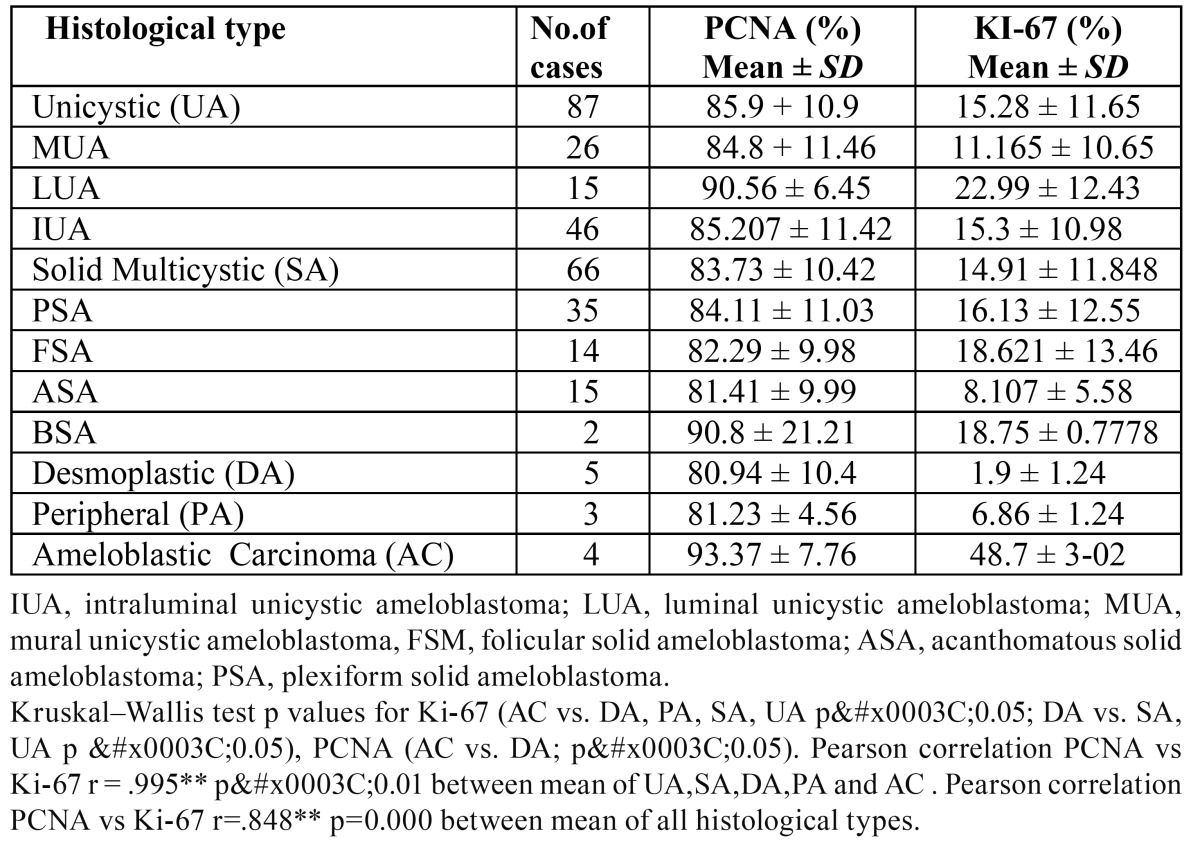
Percentages of expression of PCNA and Ki-67 for the histological types of ameloblastomas and ameloblastic carcinomas.

When analyzing cell proliferation using PCNA, we found significant differences only among the ameloblastic carcinomas (93.3%) and the DA (p<0.05), ( Table 1).

When comparing the histologic subtypes, we only observed some differences between the subtypes in the expression of both proliferation markers ( Table 1).

**Figure 1:**
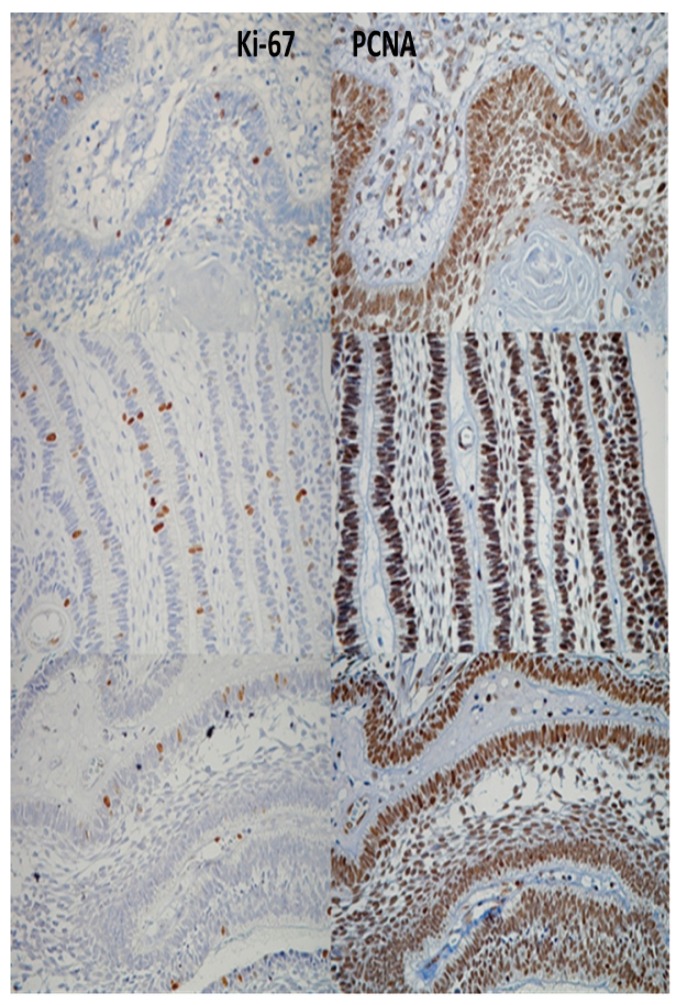
Comparative expression between Ki-67 (left column) and PCNA (right column), demonstrating a higher expression of PCNA in the same tumoral area.

When we compared the differences between the immunopositivities for PCNA and Ki-67, it was evident that the percentages were higher for PCNA in all cases (Fig. 1). In addition, the percentages were higher than 80% in all lesions, whereas the immunopositivity for Ki-67 was significantly lower; for example, AC´s expressed the greatest positivity for this immunomarker, but it only reached 48.7%, while it was 93.3% when we used PCNA.

When studying the correlation between PCNA and Ki-67, it was observed a positive and significant coefficient of correlation of Pearson r =.848 (p=0.000).

## Discussion

The usefulness of a marker for tumor diagnosis must be tested for each tumor type and application. Only those markers that have proven to be useful in practice should be considered.

The present study included a large series of ameloblastic tumors, comprising 161 ameloblastomas and four ACs, to determine the PCNA and Ki-67 positivity of each ameloblastoma variant. There are some studies addressing the apparent dispute regarding which of these cell proliferation markers are more useful—both for prognostic purposes and to understand the biological behavior of ameloblastic tumors.

In the literature, there are some studies that have used both PCNA and Ki-67 as markers of cell proliferation in ameloblastomas (21-23). However, in our study, we included a larger multicentric series of ameloblastomas and used one simple and reproducible cell-counting method (20) aiming to clarify which of the two biomarkers is more useful for indicating the proliferation of these tumors.

The term “proliferation marker” refers to specific proteins or other factors whose presence in actively growing and dividing cells serve as an indicator for such cells, for example, PCNA and Ki-67. For this type of markers, two requirements have been postulated (24):

(a) The antigen should be continuously present during the cell cycle in all cell types, and.

(b) The transition to any non-proliferative state from any part of the cell cycle should be followed by a rapid disappearance of the antigen.

In the present study, we found significant differences between some types of ameloblastomas when using the antibody Ki-67, DA was the tumor that demonstrated the lowest index of proliferation; in contrast, AC displayed the highest index of cell proliferation in comparison with all types of benign ameloblastomas (p <0.05), suggesting a more aggressive biological behavior that is characteristic of malignancy. The Ki-67 indices were similar in both UA (15.28%) and SA (14.9%), despite some differences between the various subtypes (e.g., LUA 22.99% vs. ASA 8.10%, p<0.05). This finding could be explained by the fact that this variant of ameloblastoma (LUA) contains less stellate reticulum-like cells compared with the other subtypes of UA and SA; consequently, most of the cells that were counted corresponded to the basal or suprabasal layers, which are more prone to test positive. Thus, we conclude that the proportion of the diverse types of epithelial cells, as well as the different mechanisms of growth in UA and SA, may influence the results of the proliferative index (25).

In contrast, when we used the PCNA antibody, only one significant difference was found: the AC displayed a proliferation index that was relatively higher than one ameloblastoma (DA, p<0.05). This result clearly confirmed the biological behavior of the malignant neoplasm itself. When the various subtypes of ameloblastomas were compared, no significant difference was found among them.

It should be noted that in our study, the cell proliferation index, which was expressed as the percentage of positive cells (evaluated with the labeling index), yielded much higher percentages for PCNA compared with Ki-67 in the different variants of ameloblastic tumors; this difference was evident in all the histological subtypes of ameloblastomas and AC. It is important to clarify these findings and understand why this phenomenon occurs.

PCNA is a protein that forms a ring around a portion of DNA, serving to anchor the various DNA replication and repair proteins and to regulate proliferation throughout the cell cycle. PCNA forms a toroidal, ring-shaped, 90-kDa structure by the symmetrical association of three identical monomers. The ring encircles the DNA and acts as a platform upon which polymerases and other proteins dock and perform various DNA metabolic activities (10).

This higher PCNA positivity in the nuclei of the ameloblastic tumor cells can be explained by the following factors: PCNA is an essential molecule for the synthesis of DNA; although the levels of PCNA are high when the cell enters the cell cycle, this protein has a median life of at least 20 hours within the tissues (26). This finding could indicate that nuclei can continue to express PCNA even after completing the cell cycle. In addition, the increase of PCNA levels could be induced by growth factors or as a response to damaged DNA even after the cell is no longer active in the cell cycle. PCNA is involved in the excision and replacement of abnormal nucleotides and is thus also expressed in non-proliferating cells undergoing DNA repair (24).

Other factors that might interfere with the immunoreactivity of PCNA in stored paraffinized tissues are the type of fixative used and the duration and temperature of fixation. Furthermore, the existence of cell type-specific differences between the effects of fixation and processing on the immunorecognition of Ki67 versus PCNA suggests that these antigens may be differentially affected because they are packaged differently in the various types of cells (27).

Another issue for consideration is the dilution of the antibody: the lower the dilution of antibody, the higher is the percentage of PCNA expressed in the nucleus.

It is important to note that the immunohistochemical studies for PCNA vary considerably; these differences may reflect the sizes of the samples, the numbers of cells counted per field, the cell counting techniques, and the statistical methods applied.

Therefore, all of the points mentioned above could explain the increased positivity of PCNA compared with other markers of cellular proliferation, such as Ki-67.

Some investigations have revealed that Ki-67 is not subject to the influences of internal and external factors, such as PCNA; therefore, in some studies, Ki-67 is considered to be the more specific marker of cell proliferation (11,12,17). When the Ki-67 antigen was discovered to be present in all proliferating cells (normal and tumor cells), it soon became evident thereafter that the presence of this protein is an excellent operational marker for determining the growth fraction of a given cell population. For this reason, antibodies against the Ki-67 protein are increasingly being used as diagnostic tools for various types of neoplasms.

The expression of the human Ki-67 protein is strictly associated with cell proliferation. During interphase, this antigen can be exclusively detected within the nucleus, whereas, in mitosis, most of the protein relocates to the surface of the chromosomes. The fact that the Ki-67 protein is present during all active phases of the cell cycle (G1, S, G2, and mitosis) but absent from resting cells (G0) makes it an excellent marker for determining the growth fraction of a given cell population (28).

In this study, when we used the proliferation cell marker Ki-67, the percentages obtained varied among the different types of ameloblastomas, suggesting that Ki-67 is a more specific marker for the proliferation of ameloblastic tumor cells. This difference can be attributed to Ki-67 (a protein that degrades rapidly after mitosis) because it has a lifetime of approximately 60-90 minutes (21,29).

The prognostic value of the Ki-67 index has been esta-blished in numerous publications. However, it is also evident that estimating the growth fraction alone is insufficient to describe tumor growth. For example, the growth fraction (and the Ki-67 labeling index) relates only to the number (or fraction) of proliferative cells but not to the time needed for the completion of an intermitotic cycle. In other words, the estimation of the growth fraction provides information only on the state but not on the rate of proliferation; therefore, an additional marker would be helpful to assess this parameter (28).

It should be emphasized that the molecular regulation of the cell cycle is a complex network that might include other factors rather than the expression of proteins, such as Ki-67 and PCNA. This intricate process could involve the modification (e.g., phosphorylation, ubiquitination), degradation and translocation of several key proteins.

In this paper, we clearly state that Ki-67 is a more specific proliferation marker than PCNA for ameloblastic tumors. It also should be recognized that a marker of cell proliferation can enable an understanding of how cell division works. As mentioned above, this cell division is influenced by several factors that are involved in tumor growth and tumor biology, including apoptosis, angiogenesis, tumor invasion, and tumor histomorphology.
